# Development-Specific Differences in the Proteomics of *Angiostrongylus cantonensis*

**DOI:** 10.1371/journal.pone.0076982

**Published:** 2013-10-25

**Authors:** Hui-Cong Huang, Li-Li Yao, Zeng-Mei Song, Xing-Pan Li, Qian-Qian Hua, Qiang Li, Chang-Wang Pan, Chao-Ming Xia

**Affiliations:** 1 Department of Parasitology, Medical College of Soochow University, Suzhou, Jiangsu, P. R. China; 2 Department of Parasitology, School of Basic Medical Sciences, Wenzhou Medical University, Wenzhou, Zhejiang, P. R. China; 3 Department of Laboratory Diagnosis, The Third Affiliated Hospital of Wenzhou Medical University, Ruian, Zhejiang, P. R. China; New England Biolabs, United States of America

## Abstract

Angiostrongyliasis is an emerging communicable disease. Several different hosts are required to complete the life cycle of *Angiostrongylus cantonensis*. However, we lack a complete understanding of variability of proteins across different developmental stages and their contribution to parasite survival and progression. In this study, we extracted soluble proteins from various stages of the *A. cantonensis* life cycle [female adults, male adults, the fifth-stage female larvae (FL5), the fifth-stage male larvae (ML5) and third-stage larvae (L3)], separated those proteins using two-dimensional difference gel electrophoresis (2D-DIGE) at pH 4–7, and analyzed the gel images using DeCyder 7.0 software. This proteomic analysis produced a total of 183 different dominant protein spots. Thirty-seven protein spots were found to have high confidence scores (>95%) by matrix-assisted laser desorption ionization time-of-flight mass spectrometry (MALDI-TOF MS). Comparative proteomic analyses revealed that 29 spots represented cytoskeleton-associated proteins and functional proteins. Eight spots were unnamed proteins. Twelve protein spots that were matched to the EST of different-stage larvae of *A. cantonensis* were identified. Two genes and the internal control 18s were chosen for quantitative real-time PCR (qPCR) and the qPCR results were consistent with those of the DIGE studies. These findings will provide a new basis for understanding the characteristics of growth and development of *A. cantonensis* and the host–parasite relationship. They may also assist searches for candidate proteins suitable for use in diagnostic assays and as drug targets for the control of eosinophilic meningitis caused by *A. cantonensis*.

## Introduction

Angiostrongyliasis cantonensis is a human zoonotic parasitic disease. Even before the outbreak of *A. cantonensis* that occurred in Beijing, China from June through September 2006, sporadic cases had been reported for at least sixty years. It has been reported in southeastern Asia, the south Pacific, Africa, India, the Caribbean, and, recently, Australia and North America [1]. Angiostrongyliasis is regarded as an emerging communicable disease [2], [3]. Several different hosts are required to complete the life cycle of *A. cantonensis*. Adult worms reside in normal hosts, rats, which discharge the first-stage larvae (L1) after infection. Widely disseminated via water, L1 enter an intermediate host, such as *Pomacea canaliculata*, and develop into the third-stage larvae (L3), which is the infective stage. L3 individuals infect rats, penetrate the blood-brain barrier, and develop into the fourth- and fifth-stage larvae (L4 and L5). They then develop into adults in the blood vessels of the rats' hearts and lungs. However, in some abnormal hosts (mice), after passage to the small intestine, L3 penetrate the blood-brain barrier, and then develop into L4 and L5. In infected humans, L3 may penetrate the intestinal wall. These worms fail to develop into adults and then migrate to the brain within 12 h of initial infection, producing neurological symptoms (eosinophilic meningitis) [4].

With the increase in income and living standards and the resulting pursuit of exotic and delicate foods, *A. cantonensis*-endemic areas in China are expanding rapidly. *A. cantonensis* has become the most common cause of eosinophilic meningitis in China [3], [5]. The patients all had a history of eating raw freshwater snails, which was almost certainly how they acquired *A. cantonensis*. The incubation period ranges from 1 to 36 days. The main symptoms include fever, severe headache, neck stiffness, and skin paresthesia. Laboratory examination showed a higher than normal concentration of eosinophilic granulocytes in both peripheral blood and cerebrospinal fluid [1], [6]. Although the interactions between the nematodes and their hosts are poorly understood, there is increasing concern regarding the diagnosis and treatment of eosinophilic meningitis induced by *A. cantonensis* [7], [8], [9], [10], [11].

Some investigations have shown the importance of developmental biology in several types of protozoans (such as *Plasmodium* and *Trypanosoma*) and helminthes (such as *Schistosoma*) [12], [13], [14], [15]. When parasites change hosts to complete their life cycle, they must be able to respond to the new host environment and regulate differential gene expression. In this way, a comprehensive deciphering of the proteome has become central to understanding the complex parasite-host interplay and to delivering candidate drugs and vaccine targets. The protein diversity of *A. cantonensis* has been demonstrated by SDS-PAGE. Some candidate antigens of the worms might be used in early diagnoses and epidemiological surveys of angiostrongyliasis [16]. However, the comparative proteomics were not directly identified by that approach. Two-dimensional differential gel electrophoresis (2-D DIGE) is a useful method in the study of proteomic changes. Tandem mass spectrometry is also used to identify peptide spots. We can demonstrate the value of proteomics as a tool for the identification of *A. cantonensis* proteins that are differentially expressed between various developmental stages.

The purpose of this study was to produce a deeper understanding of the characteristics of growth and development in *A. cantonensis* stages, and to use the results of this analysis to determine the developmental mechanism of *A. cantonensis*, which might also provide evidence on key parasite proteins involved in host regulation and host-parasite interactions.

## Materials and Methods

### Ethics Statement

*Pomacea canaliculata* was collected from private land in Cangnan County, Wenzhou City, in Zhejiang Province, China. Consent to collect from this private land was given by the owner who also assisted in the collection of the specimen. The owner complained that this snail was a pest on his field and appreciated the collection process. We confirm that the field studies did not involve endangered or protected species. According to the laboratory animal welfare and ethical principles, this experimental project was optimization designed and strictly planned. The protocol was approved by the Laboratory Animal Ethics Committee of Wenzhou Medical College & Laboratory Animal Centre of Wenzhou Medical College (Permit Number: wydw2010-0008). The rats were sacrificed by anesthesia with chloral hydrate. All efforts were made to minimize suffering. After the experiments, animal models were burned.

### Parasites collection

Intermediate hosts *Pomacea canaliculata* were propagated for several months in the laboratory by cycling through rat feces and more intermediate hosts to produce L3 larvae at 20 days post-infection. Infected snails were shelled and crushed. The intestines and other organs were removed and the remaining tissue was homogenized. The homogenates were filtered through a 40-mesh sieve, deposited for 5 min at 4°C, and precipitated 2–3 times at room temperature. The sediments were removed and L3 numbers and viability were determined by direct observation under a light microscope. Three-week-old female Sprague-Dawley (SD) rats (weight 100–120 g, grade clean, Certificate SYXK[ZHE] 2005-0061), supplied by the Laboratory Animal Center of Wenzhou Medical College were orally infected with 50 L3 of *A. cantonensis*/rat. The SD rats were killed at 25 days and 45 days post challenge. Then L5 *A. cantonensis* larvae were collected from the brains and the adults were collected from the blood vessels of the hearts and lungs. Individuals of different sexes were separated using morphological criteria: Females are usually longer and thinner than males, and males exhibit typical copulatory bursa. L3, L5, and adults were washed three times with 0.01 mol/L PBS buffer in order to remove residual host proteins, and stored at −80°C until further use.

### Protein extraction

Ten milligrams each of female adults (FA), male adults (MA), female fifth-stage larvae (FL5), male fifth-stage (ML5) larvae, and third-stage larvae (L3) were transferred into the homogenizer. After the addition of 400 µL DIGE lysis buffer containing 7 M urea, 2 M thiourea, 4% CHAPS, 65 mM Tris, 2% DTT, 0.2% IPG buffer (GE Healthcare, Chalfont St. Giles, Buckinghamshire, U.K.) and 0.1% v/v protease inhibitors mixture, and nuclease mixture (Merck, Darmstadt, Germany), the samples were homogenized. Then the suspensions were sonicated 5 times for 10 s each at 80 w and placed on ice during 15 s rest intervals. Then the samples were centrifuged at 12,000 rpm and 4°C for 45 minutes to collect the supernatant. The supernatant was filtered with 0.22 µm filter to obtain clear samples. The total protein concentration was determined with Bio-Rad protein assay reagent and stored at −80°C until use in proteomic analysis.

### 2-D DIGE

The pH of each protein sample was adjusted to 8.5 with 50 mmol/L NaOH solution. Equal amounts (50 µg) of each of the 5 samples were labeled with 400 pmol fluorescent dye. For each stage, two independent sample preparations were analyzed by 2-D DIGE. The reference standard was a mixture of 5 equal protein samples, which was dissolved in freshly prepared dimethylformamide. Cy2 was always used to label the reference standard and Cy3 and Cy5 were alternated to avoid artifacts due to preferential labeling (CyDye DIGE Fluors, GE Healthcare). Combining samples minimizes gel-to-gel variations and allows for pair-wise comparisons in a single gel with different labels. The proteins were labeled for 30 min on ice in darkness. The labeling reaction was quenched by incubation with 1 µL of 10 mM L-lysine (GE Amersham Biosciences) for 10 min on ice in the dark.

Samples labeled with fluorescent dye were applied to nonlinear IPG strips (13 cm long, pH 4–7) (GE Healthcare) on the rehydration tray and focused using an Ettan IPGphor Isoelectric Focusing System (GE Amersham) as follows: active rehydration at 30 V for 12 h, followed by isoelectric focusing for a total of approximately 60 kVh (step to 500 V for 1 h, step to 1000 V for 1 h, step to 8000 V for 8 h, step to 500 V for 4 h). After isoelectric focusing, disulfide bonds were reduced by placing the IPG strips in equilibration buffer I for 15 min (6 M urea, 2% SDS, 50 mM Tris-HCl, pH 8.8, 30% glycerol, 0.002% Bromophenol blue, 10 mg/mL DTT). Then the strips were incubated for 15 min in equilibration buffer II (6 M urea, 2% SDS, 50 mM Tris-HCl, pH 8.8, 30% glycerol, 0.002% Bromophenol blue, 400 mg iodoacetamide). For the second dimension, the IPG strips were placed on. 12.5% polyacrylamide gels in an Ettan Dalt 6(GE Healthcare) electrophoresis system at 20°C. Electrophoresis parameters were 15 mA/gel 30 min and 30 mA/gel. The reaction was stopped after approximately 6 h. A running buffer of 25 mM Tris, pH 8.3, 192 mM glycine, and 0.1% SDS was used.

### Gel imaging and gel data analysis

Individual fluorescent images of Cy2-, Cy3-, and Cy5-labeled proteins from each gel were obtained using UMax Powerlook 2110XL (GE Amersham) and Typhoon FLA9000 (GE Amersham) with excitation and emission wavelengths of 488 and 520 nm for Cy2, 532 and 580 nm for Cy3, and 633 and 670 nm for Cy5. Image analysis was performed using DeCyder 7.0 software (GE Healthcare) to identify proteins displaying differential expression levels. Statistical analysis and gel-to-gel comparison were performed with a DeCyder Differential In-gel Analysis and DeCyder Biological Variation Analysis (BVA) software module. The estimated number of spots for each codetection procedure was set at 2500. Proteins were detected and quantified, gels were matched, and numbers of protein expression spots were determined. The gel with the highest spot count was designated the master gel. Protein expression values were statistically analyzed using Student's *t* test, as were selected protein spots for which AR>2.0. *P*<0.05 was considered statistically significant. Those spots were chosen as suitable for attempted protein identification using matrix-assisted laser desorption/ionization time of flight mass spectrometry (MALDI-TOF MS) after this comprehensive examination.

### Spot excision and identification

After imaging, protein extracts were separated on preparative gels and proteins of interest were recovered from the gels for identification. Proteins (800 µg) from each sample were resolved on separate preparative polyacrylamide gels and visualized by staining with Coomassie brilliant blue (Bio-Rad, Hercules, CA, U.S.). All the differentially expressed spots were selected and excised manually from the preparative gels. The specific methods of in-gel tryptic digestion (trypsin, 20 hours), extraction of enzyme peptides, ZipTip desalting, MALDI-TOF MS, analysis, and protein identification are described in detail below.

### In-gel tryptic digestion and ZipTip desalination

After excision, particles were cut into smaller pieces, destained by adding 300 µL 100 mM NH_4_HCO_3_ in 30% acetonitrile (ACN). After removing the destaining buffer, the gel pieces were lyophilized and rehydrated in 30 µL of 50 mM NH_4_HCO_3_ containing 50 ng trypsin (sequencing grade; Promega, Madison, WI, U.S.). The mass ratio of enzyme and protein was generally 1∶40. After overnight digestion at 37°C, the hydrolyzate was absorbed and transferred to a new tube. The peptides were extracted three times with 60% ACN in 0.1% trifluoroacetic acid (TFA), combined with the previous solution, and then freeze-dried. The resulting lyophilized tryptic peptides were kept at −80°C until mass spectrometric analysis. A protein-free piece of gel was treated as above and used as a control. ZipTip (Millipore, U.S.) desalination was performed if the products contained salt.

### MALDI-TOF MS analysis

After digestion and freeze-drying, the samples were reconstituted in 2 µL standard diluent (20∶80 ACN∶water) and spotted on a 384-well Opti-TOF stainless steel plate, covered with 0.5 µL 5 mM cyano-4-hydroxycinnamic acid (CHCA) matrix solution (solvent 50% ACN, 0.1% TFA). MS and MS/MS data were used for protein identification. They were obtained using a MALDI-TOF-TOF instrument (ABI 4800 proteomics analyzer; Applied Biosystems, Foster City equipped with a Nd:YAG 355 nm laser). Instrument parameters were set using the 4000 Series Explorer software (Applied Biosystems). Peptide mass maps were acquired in a positive ion reflector mode (20 kV accelerating voltage). Monoisotopic peak masses were automatically determined within the mass range 800–4000 Da with a signal-to-noise ratio minimum set to 10 and a local noise window width of m/z 250. 8 of the most intense ions were selected as precursors for MS/MS acquisition, excluding common trypsin autolysis peaks and matrix ion signals. In MS/MS positive ion mode, collision energy was 2 kV, collision gas was air, and default calibration was set by using the Glu1-Fibrino-peptide B ([M+H]+ 1,570.6696). The MS and MS/MS spectra were searched against the UniprotKB/SwissProt database using MASCOT version 2.2 (Matrix Science, Ltd.).

### Bioinformatics analysis and gene ontology analysis

The MS and MS/MS spectra were evaluated against the MASCOT search engine (http://www.matrixscience.com) embedded in GPS-Explorer software 3.6 (Applied Biosysterm) to identify proteins from non-redundant databases (http://www.ncbi.nlm.nih.gov/BLAST). The MALDI data and protein annotations(downloaded in March 1 2013) were also manually verified against the GenBank translated protein database as follows: Database (NCBI); taxonomy, *Caenorhabditis elegans, Caenorhabditis briggsae*, EST_*Caenorhabditis elegans*, EST_*Angiostrongylus cantonensis*, proteins from *Angiostrongylus cantonensis*, and other nematodes; type of search, peptide mass fingerprint, MS/MS ion search; enzyme, trypsin; fixed modifications, carbamidomethyl [C]; mass values, monoisotopic; protein mass, unrestricted; peptide mass tolerance (±100 ppm); fragment mass tolerance (±0.8 Da); peptide charge state (1+); maximal missed cleavages (1); GPS Explorer protein confidence index above 95% were used for further manual validation.

We used a local blastp program (version 2.2.23+) to search identified proteins against UniProt Knowledgebase Release 2013_03 consists of Swiss-Prot and TrEMBL databases. A text file which consisted of each identified protein's accessions number and the corresponding protein's accessions was contained from blastp output of Swiss-Prot and TrEMBL database with alignment> = 30% and E-value<1e-10 used an in-house Perl script to. Then, base on this text file and gene association file from gene ontology ftp, each identified protein's accessions and the corresponding GO terms were confirmed using an in-house Perl script.

### Quantitative real-time PCR verification

Total RNA was isolated from *A. cantonensis* at different developmental stages (FA, MA, FL5, ML5, and L3) using a RNeasy Mini Kit as recommended by the manufacturer (Qiagen, Germany). The ratio of absorbance at 260 nm to 280 nm (A260/A280ratio) was used to assess the purity of RNA samples using the UV-2802H spectrophotometer (Unico, China). RNA integrity and concentration were assessed, electrophoresed on 2% agarose gel, and evaluated using Quantity One 1-D Analysis Software 4.62 (Bio-Rad, U.S.).

Total RNA was reverse-transcribed into single-strand cDNA with M-MLV reverse transcriptase and oligo dT(18) primers (Qiagen, Germany) according to the manufacturer's instructions. The reaction mix was incubated for 5 min at 95°C, followed by 30 min at 42°C and 15 min at 72°C. It was then held at 4°C. The cDNA samples were then stored at −80°C for quantitative PCR.

Quantitative real-time PCR was performed in 20 µl volumes with SYBR Premix Ex Taq™ II (Takara, Japan) at 95°C for 30 s followed by 40 cycles of 95°C for 5 s and 60°C for 34 s for amplification and then 95°C for 15 s, 60°C for 1 min, 95°C for 30 s, and 60°C for 15 s for melt curve using an ABI 7500 Fast Real-Time PCR system (Applied Biosystems, Warrington, U.K.). The *A. cantoneniss* 18S ribosomal RNA gene (GenBank: AY295804.1) served as an internal control. ΔΔCt was calculated for each sample, and the expression levels were indicated with 2-ΔΔCt using the ABI 7500 Software v2.0.1(Applied Biosystems, U.S.).The PCR products were electrophoresed on 2% agarose gel and detected using ethidium bromide staining. Primer specificity was tested using a BLAST analysis against the genomic NCBI database. Primer information, including sequences and product sizes, is summarized in Table 1.

**Table 1:** Primer sequences and amplicon lengths of qRT-PCR products of target genes.

Spot	Target gene	Primers	Amplicon length (bp)
609	ACL3_0014_G09 Angiostrongylus cantonensis third stage larvae cDNA library mRNA sequence Angiostrongylus cantonensis cDNA,	FP: 5′GCACGTCGCAAATGAAGCCCA 3′ RP:5′ CGGAGAGGTGTCCGGCATCGT 3′	114
1004	Angiostrongylus cantonensis galectin 1 mRNA, complete cds	FP: 5′ ATCCGTGCCCATGACGACCG 3′ RP:5′ ACCGGTTGCGATTCCGCTCT 3′	183
	Angiostrongylus cantonensis 18S small subunit ribosomal RNA gene, as an internal control	FP: 5′ TTCGAGTATCCAGTGGAGGG 3′ RP:5′ GCAAATGCTTTCGCTTTAGG 3′	405

Transcript levels were normalized relative to those of 18S small subunit ribosomal RNA.

## Results

### Stage-specific samples

**Figure 1:**
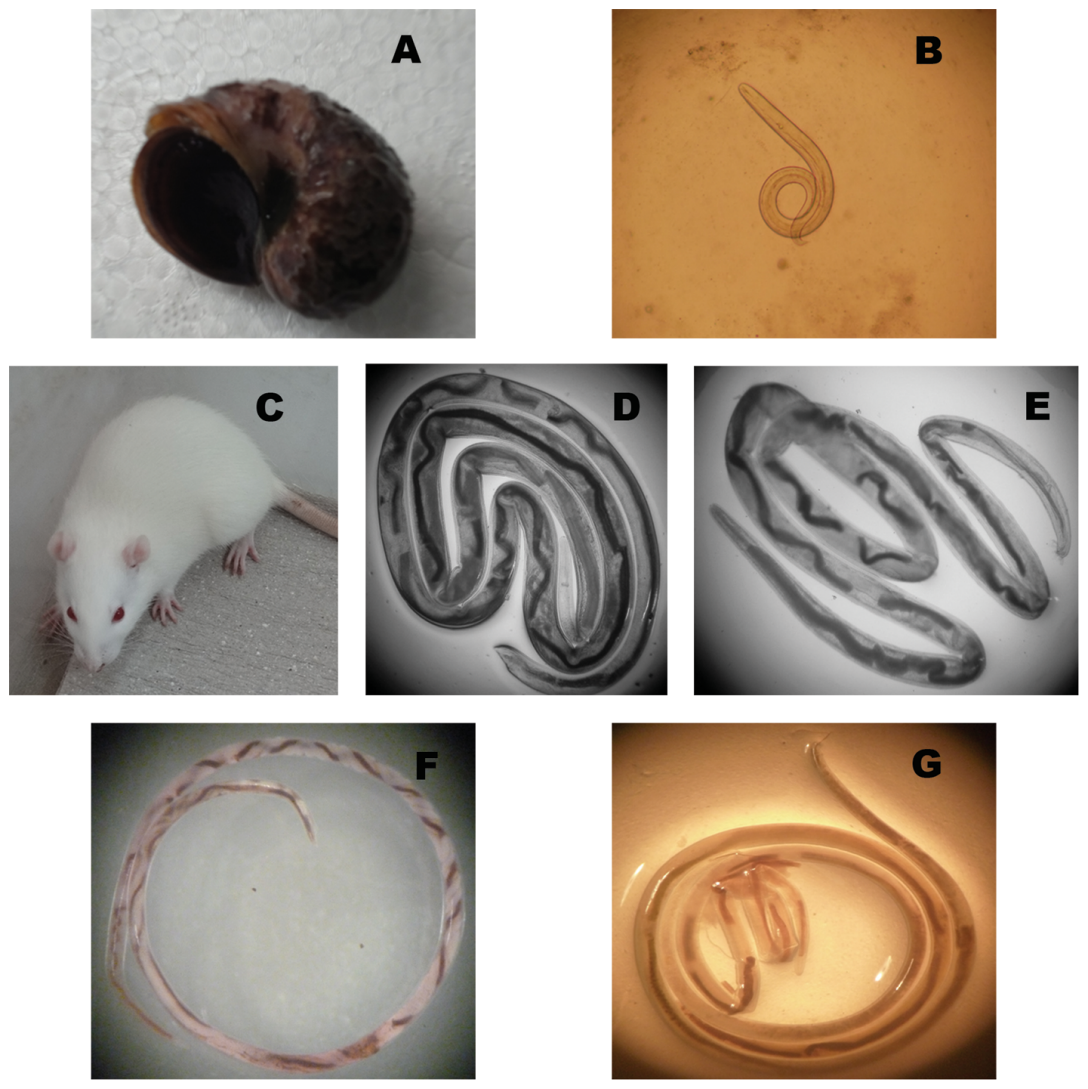
Different developmental stages of *Angiostron gylus cantonensis* and their hosts. **A**, *Pomacea canaliculata*, as the intermediate host. **B**, the third stage larvae (L3) (10×10). **C**, Sprague-Dawley (SD) rats, as the difinitive host. **D**, female of the fifth stage larvae (FL5)(10×4). **E**, male of the fifth stage larvae (ML5)(10×4). **F**, female of the adult (FA)(10×). **G**, male of the adult (MA)(10×).

The stage-specific proteome depends on sample purity. Strategies collected from previously published studies were used to obtain individual parasites from the correct stage using the specific characteristics of each stage of the life cycle. L3 was only morphologically recognizable when extracted from *Pomacea canaliculata* (Figure 1A and 1B). Purified L5 and adults were taken solely from rats (Figure 1C), at 25 days after infection for L5 and at 45 days for adults. Acquisition sites were strictly delimited as well: brains for L5 (Figure 1D and 1E) and the blood vessels of the heart and lung bloods for adults (Figure 1F and 1G). We selected younger rats as a model to ensure the success rate of infection and worm recovery.

Average protein concentrations, which was determined using Bio-Rad protein assay reagent were 11.203 µg/µl, 18.996 µg/µl, 19.217 µg/µl, 13.903 µg/µl, and 11.891 µg/µl for FA, MA, FL5, ML5, and L3, respectively. Each sample was subjected to 2-D DIGE.

### 2-D DIGE analysis of differentially expressed proteins

**Figure 2:**
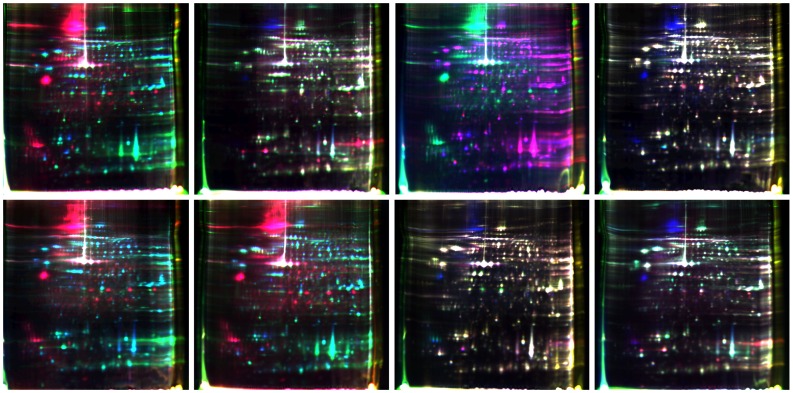
2D-DIGE gels illustrating proteins of five developmental stages of *A.C*. In standard conditions, internal control samples containing the proteins of mixture of 5 equal protein samples labeled with Cy2 (blue). Cy3 (green) and Cy5 (red) were alternated. Color picture was the overlapping images.

To assess changes, the relative abundances of proteins in five samples were quantified on 2-D DIGE gels. After labeling and fractionation by DIGE, the reference gel (Figure 2) resolved 1,395–1,768 protein spots/gel, which were then compared across the pairwise gel images (Table S1 in File S1). Only spots with statistically significant values were examined across all 24 images for reproducibility. Clear correlations to the pick gel images were considered for further analysis.

**Figure 3:**
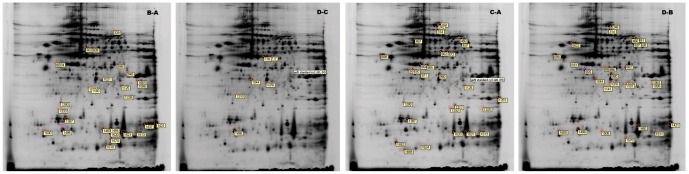
2-DG gels of total protein extracts from various stages of *A.C*. Bottom: Comassie blue-stained gels illustrating differentially regulated spots in pairwise samples of FA (A), MA (B), FL5(C) and ML5 (D) of *A.C*, as revealed by quantitative and statistical analyses with DeCyder software.

**Figure 4:**
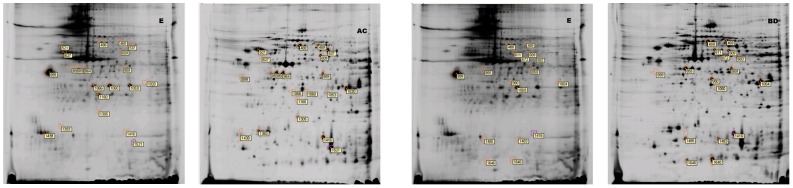
2-DG gels of common extracts from FA(A)/FL5 (C) and MA (B)/ML5(D) and L3 of *A.C.* Bottom: Comassie blue-stained gels illustrating differentially regulated spots common existence in FA(A)/FL5 (C) and MA(B)/ML5 (D), were examined for pairwise differences to the L3(E).

The distribution and relative intensity of protein spots was consistent across all groups, and background subtraction, quantification, normalization, and gel matching were fully automated. When we compared the protein spots in pairwise samples of FA/MA, FL5/ML5, FA/FL5, and MA/ML5, 49 spots were found to differ significantly (*P<0.05*). These are shown in Figure 3 and Table S2 in File S1 (AR>2.0). There was a vast difference between L3 and the other four developmental stages. Proteins common in FA/FL5 and MA/ML5, were examined for pairwise differences from L3 and selected for suitability for protein identification. A total of 170 spots were found to differ significantly. These are shown in Figure 4 and Table S3 in File S1 (*P<0.05*) (AR>2.0). Thirty-six spots were commonly existed both Figure 3 and Figure 4. In this way, a total of 183 different dominant protein spots were expressed.

Twenty-three protein spots were found to be differentially expressed between the FL5 and the FA. Of these, 15 were up-regulated in FL5 relative to FA and 8 were down-regulated. Twenty-one protein spots were found to be differentially expressed between ML5 and MA. Of these, 10 were up-regulated in ML5 relative to MA and 11 were down-regulated. Spots 314, 537, 698, 925, and 980 were all up-regulated in L5 than in adults but showed no significant differences between males and females. Eighty-nine spots were up-regulated in L5 and adults relative to L3. In contrast, 13 spots were up-regulated in L3. The combination of these effects may lead to the significant morphological differences observed between developmental groups. The variability observed in some sample groups probably reflects gender differences. Twelve spots were up-regulated and 7 spots were down-regulated in MA relative to FA. Three spots were up-regulated in FL5 and 3 spots were up-regulated in ML5. Spot 1496 was up-regulated in both ML5 and MA relative to FL5 and FA, respectively, suggesting a difference between the sexes.

### Identification and interpretation of differentially expressed proteins

Sixty-eight protein spots were considered suitable for downstream analysis by MALDI MS/MS, based on the amount of protein, matching across gels, and the presence of the protein spot on the preparative gels. Out of these, 37 proteins were successfully identified with high confidence (>95%) scores by MS. These are listed in Table 2. Among the predicted proteins, 29 spots were cytoskeleton-associated proteins and functional proteins, such as actin, paramyosin, heat shock protein, and transformation domain-associated protein (TRR-1). Their functions include metabolic processing, immune reaction, and transcriptional regulation. Eight spots represented unnamed proteins. No nuclear genomes for *A. cantonensis* are available in the NCBInr database. In this way, most of the uninterpreted experimental tandem spectra were matched to predicted fragment patterns from homologous species (e.g., the model organism *Caenorhabditis elegans*). Although 12 spots were matched to the EST of different-stage larvae of *A. cantonensis*, they could not be identified. These functions probably correspond to genes that have yet to be described (Table 3). Their peptides information is shown in Table S4 in File S1. Several spots were attributable to the same peptides information. Protein DIM-1 isoform, for example, was found in spots 850, 855, 864, and 865.

**Table 2:** Uniquely identified protein spots from FA, MA, FL5, ML5 and L3.

Spot	Protein ID	GI	Species	Protein MW	PI	Pep Count	Seq. Coverage %	Protein Score	Protein Score C.I.%
246	paramyosin	gi|6896	*Caenorhabditis elegans*	102002.2	5.28	19	21	181	100
314	hypothetical protein	gi|256251592	*Angiostrongylus cantonensis*	37942.2	6.42	13	33	209	100
451	unnamed protein product	gi|259437037	*Caenorhabditis elegans*	82111.4	8.55	13	14	62	96.05
467	Protein TNT-2, isoform a	gi|71991390	*Caenorhabditis elegans*	48712	4.87	11	17	139	100
488	putative Heat Shock Protein	gi|256251570	*Angiostrongylus cantonensis*	44269	4.99	20	45	414	100
522	protein disulphide isomerase	gi|94442975	*Caenorhabditis briggsae*	55347	4.73	12	17	199	100
537	protein disulfide isomerase 1	gi|20068287	*Ostertagia ostertagi*	55314	6.7	7	14	133	100
608	Protein Y51H1A.2, isoform e	gi|392892365	*Caenorhabditis elegans*	39393	7.43	12	29	63	96.87
627	Protein B0546.3	gi|351018258	*Caenorhabditis elegans*	57384	5.58	12	20	65	97.98
662	hypothetical protein	gi|256016687	*Angiostrongylus cantonensis*	44224	6.05	18	53	488	100
672	hypothetical protein	gi|256016687	*Angiostrongylus cantonensis*	44224	6.05	19	58	488	100
698	Protein CALU-1, isoform a	gi|71994129	*Caenorhabditis elegans*	36169.1	4.64	6	17	124	100
717	actin-1	gi|329669006	*Angiostrongylus cantonensis*	26138	5.25	9	33	247	100
730	actin-1	gi|329669006	*Angiostrongylus cantonensis*	26138	5.25	9	42	248	100
848	galectin 5	gi|341864437	*Angiostrongylus cantonensis*	36104	7.08	12	32	68	99.99
850	Protein DIM-1, isoform a	gi|351050469	*Caenorhabditis elegans*	72375	8.11	8	13	169	100
855	Protein DIM-1, isoform a	gi|351050469	*Caenorhabditis elegans*	72375	8.11	9	13	233	100
859	Hypothetical protein CBG04457	gi|268552921	*Caenorhabditis briggsae*	37211	6.51	10	28	70	99.77
862	Protein TNI-3	gi|3879965	*Caenorhabditis elegans*	29877	5.02	8	15	80	99.93
864	Protein DIM-1, isoform a	gi|351050469	*Caenorhabditis elegans*	72375	8.11	8	12	248	100
865	Protein DIM-1, isoform a	gi|351050469	*Caenorhabditis elegans*	72375	8.11	8	12	262	100
885	Protein TNI-3	gi|3879965	*Caenorhabditis elegans*	29877	5.02	9	21	93	100
914	Hypothetical protein CBG14099	gi|268578649	*Caenorhabditis briggsae*	30614.4	9.2	2	6	85	99.99
925	galectin 1	gi|341864429	*Angiostrongylus cantonensis*	32720	6.32	7	20	41	97.43
945	Protein NEX-1	gi|17554342	*Caenorhabditis elegans*	35730.6	6.13	5	22	185	100
1004	galectin 1	gi|341864429	*Angiostrongylus cantonensis*	32720	6.32	16	42	449	100
1020	galectin 2	gi|341864431	*Angiostrongylus cantonensis*	31731	6.07	17	51	426	100
1060	actin-1	gi|329669006	*Angiostrongylus cantonensis*	26138	5.25	1	4	54	99.86
1063	galectin 2	gi|341864431	*Angiostrongylus cantonensis*	31731	6.07	20	62	253	100
1141	Protein PAS-5	gi|3876333	*Caenorhabditis elegans*	27247.1	5.27	9	40	250	100
1253	Thioredoxin peroxidase	gi|46576851	*Ascaris suum*	21690	6.21	5	26	88	99.99
1305	actin depolymerizing factor homolog unc-60 -	gi|7494523	*Caenorhabditis elegans*	32957.6	6.36	6	17	133	100
1383	putative Heat Shock Protein	gi|256016555	*Angiostrongylus cantonensis*	18039	5.06	9	47	198	100
1421	putative Lipid Binding Protein	gi|256016483	*Angiostrongylus cantonensis*	19042	6.92	4	30	54	99.87
1520	indoleamine dioxygenase like-myoglobin	gi|3582430	*Batillus cornutus*	41787	6.47	10	22	63	96.18
1521	Protein TRR-1, isoform c	gi|71984162	*Caenorhabditis elegans*	469066	6.23	29	8	67	98.69
1640	Nematode Polyprotein Allergen related family member(npa-1)	gi|72000798	*Caenorhabditis elegans*	138698	6.98	21	16	63	97.00

**Table 3:** Unidentified protein spots match to the EST of *A. cantonensis*.

Spot	Protein	GI	Protein MW	PI	Pep. Count	Seq. Coverage %	Protein Score	Protein Score C.I.%
609	ACL3_0014_G09	gi|342297740	21662.8	9.38	6	40	63	99.17
611	ACL5_13G01	gi|60292955	22079.1	9.32	1	6	69	99.82
980	ACL5_06F14	gi|60292360	26334.3	8.27	3	12	74	99.93
1044	ACL5_06F14	gi|60292360	26334.3	8.27	8	27	258	100
	ACL5_10D24	gi|60292678	24559.7	9.25	8	29	253	100
1076	ACL5_01F07	gi|60291966	23054.7	8.65	8	38	350	100
	ACL5_06F14	gi|60292360	26334.3	8.27	8	33	344	100
	ACL5_10D24	gi|60292678	24559.7	9.25	8	29	334	100
1416	ACL5_06F20	gi|60292366	24320.1	5.86	8	38	222	100
	ACL5_13H09	gi|60292972	25638.9	5.98	8	36	221	100
	ACL5_14A04	gi|60292978	23494.9	6.12	8	40	197	100
1437	ACL5_06F20	gi|60292366	24320.1	5.86	6	25	84	99.99
1460	ACL5_02H23	gi|60292080	24813.9	8.36	10	32	187	100
	ACL5_02C14	gi|60292018	26913.1	8.31	10	29	184	100
1511	ACL5_10H15	gi|60292715	23739.3	6.31	10	36	208	100
	ACL5_02H23	gi|60292080	24813.9	8.36	10	34	204	100
	ACL5_02C14	gi|60292018	26913.1	8.31	10	31	201	100
1513	ACL5_12M13	gi|60292849	22307.5	5.5	11	36	158	100
	ACL5_10H15	gi|60292715	23739.3	6.31	11	34	146	100
	ACL5_02H23	gi|60292080	24813.9	8.36	11	33	140	100
	ACL5_02C14	gi|60292018	26913.1	8.31	11	30	137	100
1570	ACL5_12M13	gi|60292849	22307.5	5.5	7	26	97	100
1592	ACL5_01B08	gi|60291922	25750.6	8.44	10	33	364	100

**Figure 5:**
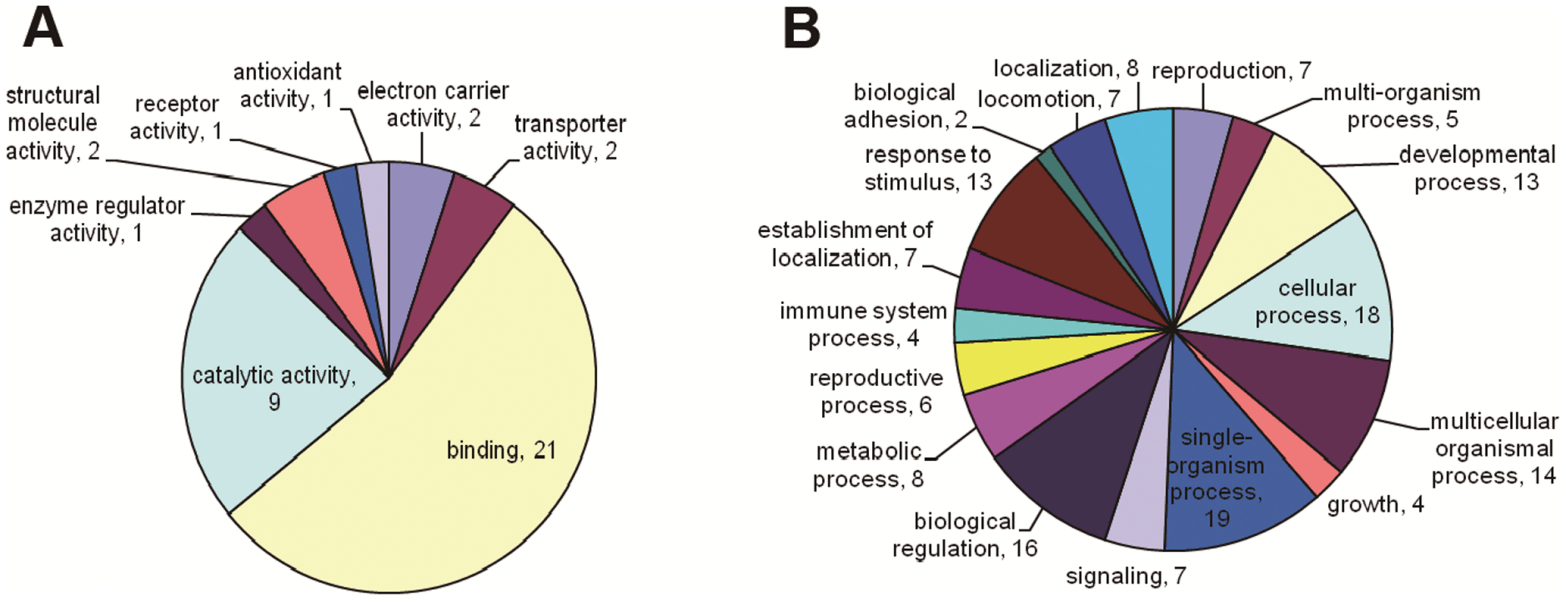
Distribution of Gene Ontology terms (level 2) for protein identified in differential expressed in FA, MA, FL5, ML5 and L3 stage of *A.C* nematodes. **A**: Moledular Function **B**: Biological Process.

The GO based data illuminate the different functions and processes in which the proteins identified in the differentially expressed proteins (DEP) are putatively involved. In the total set of DEP and the individual subsets, binding (GO: 0005488) was the major molecular function categories. The major biological process categories include single-organism process (GO: 0044699), cellular process (GO: 0009987) and biological regulation (GO: 0065007). These are shown in Figure 5.

### Quantitative real-time PCR analysis of differentially expressed proteins

**Figure 6:**
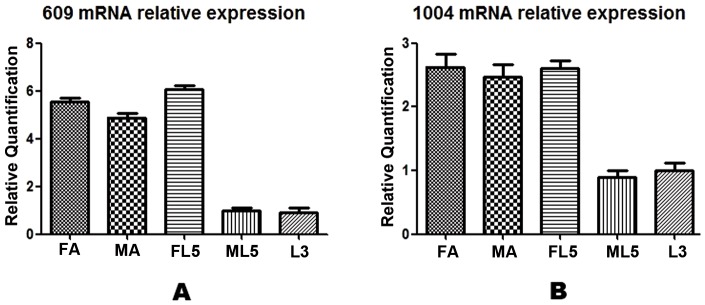
Quantitative Real-time PCR Analysis of Differentially Expressed Proteins of *A.C* nematodes. **A**: The expression of 609 increased to 6.11 fold in FL5 and 5.58 fold in FA, 4.95 fold in MA *(P<0.01)*, but there was little change in ML5 *(P>0.05)*. **B**: The expression of 1004 increased to 2.60 fold in FL5 and 2.62 fold in FA, 2.50 fold in MA *(P<0.01)*, but there was little change in ML5 *(P>0.05)*.

Five RNA samples gave excellent purity values with OD260/280 between 1.9 and 2.2. Two genes corresponding to protein spots designated 609 and 1004 and the internal control 18s were chosen for quantitative real-time PCR (qPCR) analysis to quantify their levels of transcription. The fold change of the expression of the proteins in these 2 spots in L5 and adult worms were calculated and normalized by the level of expression at L3. The expression of the protein in spot 609 increased 6.11 fold that of L3 in FL5, 5.58 fold in FA, and 4.95 fold in MA (*P*<0.01), but there was little difference between ML5 and L3 (*P*>0.05). We also found that the expression of the protein in spot 1004 increased to 2.60 fold that of L3 in FL5, 2.62 fold in FA, and 2.50 fold in MA (*P*<0.01), but there was little difference between ML5 and L3 (*P*>0.05) (Figure 6). The qPCR results were consistent with those of the DIGE studies, suggesting that the proteins here identified as differentially expressed were regulated at the transcriptional level.

## Discussion

*Angiostrongylus cantonensis*, which is a parasite from the phylum Nematoda, can lead to serious public health problems. Prevention and control of this foodborne zoonotic disease is a matter of considerable importance, and for this preventing infection is the most essential step [17], [18]. However, *A. cantonensis* infection can take place not only by ingestion of intermediate hosts but also by ingestion of vegetables and drinking water and through hand-to-mouth contact after touching a contaminated object [3], [19]. Current methods used to reduce the impact of *A. cantonensis* infection rely heavily on the professional knowledge of healthcare workers to ensure timely detection of individual infections and adequate medical response [20], [21]. Biological research, such as studies into the genomics, functional genomics, and proteomics of this organism can help clinicians improve diagnostic techniques and treatments for Angiostrongyliasis cantonensis. Few studies have employed *A. cantonensis* proteomic approaches to study their biology due to the scarcity of genomic information available. Genome research was certainly one of the most important scientific disciplines. As technologies improve and costs continue to decrease, complete genomes will be gradually become more available and will eventually be taken for granted by researchers as an obvious piece of core knowledge for any organism under study [22]. Proteomics screenings have been useful in pinpointing known proteins; studies of protein structure, function, and interactions have become a new paradigm for investigating basic biology and developing therapeutics. The nematode *Caenorhabditis elegans* is the best-studied model organism [23], [24]. Although molecular mass extremes proteins, pH extremes proteins and hydrophobic proteins will be missed by the extraction protocol, 2-D DIGE can circumvent the gel-to-gel variability inherent in conventional 2-DE and is particularly useful in the study of proteome changes in applications such as developmental biology and tissue proteomics [25]. The present study resulted in the identification and measurement several protein differences between different stages of the *A. cantonensis* life cycle. They are here expressed as pairwise fold changes, as determined using mass spectrometry. We expect that pathways involved in the developmental changes of the various stages of *A. cantonensis* will be revealed in later studies.

Differential expression of protein spots was detected and evaluated in pairwise samples of FA/MA, FA/FL5, MA/ML5, FL5/ML5, FA and FL5/L3, and MA and ML5/L3. Potentially implicated in gender-related and age-related changes. First of all, in this paper, we identified the proteins likely to be involved in development, focusing on functional proteins. Several proteins involved in metabolism were found to differ across adults and L5. Among these, protein Disorganized muscle-1 (DIM-1) and protein disulfide isomerase-1 (PDI) were decreased in FA and MA, respectively. Furthermore, several proteins related to reproductive system, such as Thioredoxin peroxidase (TPx) was increased in FA relative to FL5. DIM-1, which encodes components of muscle attachment structures, was identified as a novel immunoglobulin superfamily protein in *C. elegans*, localized to the region of the muscle cell membrane around and between the dense bodies that anchor the actin filaments. These proteins may play a role in stabilizing the thin filament components of the sarcomere. Recently finding that *dim-1* has reduced maximal bending indicates a new function for *dim-1* in nematode locomotion. Loss of DIM-1 protects against attachment complex disruption and resultant intramuscular pathologies [26], [27], [28]. PDI is a member of the thioredoxin superfamily of thiol/disulfide exchange catalysts. It is a good oxidase because of the high level of reactivity between the disulfide and the two active site cysteines [29]. Here, it was demonstrated that PDI was up-regulated in FL5, but the specific disulfide targets were not identified. TPx is a member of the peroxiredoxin family. It plays a dominant role in a hydrogen peroxide metabolism, which regulates the levels of reactive oxygen species in cells and tissues and protects them from oxidative damage [30]. Immunolocalization studies indicated that the expression of TPx is ubiquitous in *O. viverrini* organs and tissues. It was also detected in bile fluid and bile duct epithelial cells surrounding the flukes after infection. In this way, TPx plays a significant role in protecting the parasite from damage induced by reactive oxygen species produced by inflammation [31]. The increased thioredoxin peroxidase in FA suggests an increase in the antioxidant capacity in these FA over FL5. Levels of galectin 1 and troponin I 3(TNI-3) were elevated 11.00-fold and 2.37-fold higher, respectively, in ML5 than in MA, as indicated by DIGE analysis. Galectin is a lectin that can bind to galactoside. It is found in different tissues in many different types of organisms. The galectin family has 15 members, and participates in many physiologic and pathologic processes, including cell adhesion, cell growth regulation, RNA post-transcriptional processing, inflammation, immune regulation, tumor transformation, and cell apoptosis [32], [33], [34]. The deduced amino acid sequences of the TNI isoforms of *C. elegans* were found to be involved in inhibition of the actomyosin ATPase activity. There are four TNI genes encoding four different isoforms in *C. elegans*. The effects of interfering with isoform function using isoform-specific RNAi constructs shows that the *tni-1* and -*2* genes produce abnormal locomotion; RNAi treatment interfering with *tni-3* function led to abnormal muscle morphology, egg laying defects, and constipation; *tni-4* function is required in completion of normal pharynx formation [35], [36]. In contrast, levels of galectin 2 and protein TNI-3 were approximately 5.00-fold and 2.72-fold higher, respectively, in MA than in ML5. It is likely that phosphorylation, glycosylation modification, and transcriptional regulatory mechanisms participate in that process. There is some question as to whether they influence each other during the growth and development. Our first conclusion that can be made from the existence of such diversity was detected using protein expression techniques and changes in various developmental stages. The proteins described above may serve as targets for further investigations. Among the vast number of proteins whose expression levels differ between L3 and the other developmental stages, actin-1 was the most abundantly expressed in L3 relative to the other four groups. This is consistent with the active twist in L3. As L3 is an infective stage, this could indicate that high levels of actin-1 expression may be responsible for the infectivity of L3 [37].

Secondly, about 48% (12/25) of the differentially expressed proteins were identified in pairwise samples of FA/MA and FL5/ML5. The up-regulation of numerous proteins may be involved in immune invasion in females. Indoleamine dioxygenase-like-myoglobin (IDO; spot 1520) can bind oxygen reversibly. Because it is a tryptophan-degrading enzyme, IDO is required for the catabolism of the aromatic amino acid L-tryptophan and the generation of L-tryptophan metabolites. L-tryptophan and IDO-independent metabolites of tryptophan have been shown to activate proinflammatory Th17 cells [38]. Because it is an immune privilege-associated enzyme, IDO has also been studied in mammalian pregnancy, tumor resistance, chronic infection, and autoimmune disease [39], [40], [41]. The high levels of expression of NEX1 annexin and members of the TRRAP-like family suggests that these proteins are involved in specific functions. Annexins are structurally related proteins that bind phospholipids in a calcium-dependent manner. There are 4 *C. elegans* annexins, nex-1, -2, -3 and -4, throughout the development. The protein is associated with membrane systems of the secretory gland cells of the pharynx, with sites of cuticle formation in the grinder in the pharynx, with yolk granules in oocytes, with the uterine wall and vulva, and with membrane systems in the spermathecal valve. It also can mediate apoptotic cell engulfment. In the nematode *C. elegans*, down-regulation of the annexin homolog prevents efficient engulfment of pharyngeal cell corpses [42], [43], [44]. Transformation/transcription domain-associated protein (TRRAP) has been found to correlate with *C. elegans* vulval development, which also appears to be responsible for recruitment of histone acetyltransferase (HAT) enzymes and the coordination of distinct chromatin-based processes [45], [46]. The down-regulation of IDO, NEX1 annexin, and members of the TRRAP-like family observed in adult male *A. cantonensis* and the up-regulation observed in adult female *A. cantonensis* was coincidence with those published data. It may be related to the observation that female adults survived better than the male adults [47], [48]. However, these proteins were not found to be up-regulated in FL5 compared to ML5. The expression of galectins, a family of soluble beta-galactosyl-binding lectins, showed increased expression in the FA relative to MA. This was believed to mediate cell-cell and cell-extracellular matrix interactions during development, inflammation, apoptosis, and tumor metastasis [49], [50]. Since lipid metabolism, maintenance of the proteasome, insulin signaling and nuclear pore complexes are essential for germline deficient phenotypes [51]. The expression of proteasome core alpha subunit 5 (PAS-5) and putative Lipid Binding Protein showed increased expression both in the MA relative to FA and in MA relative to ML5, which are related to gender-specific development of *A.cantonensis*. The catalog of proteins reflects different gender-specific related processes of immune evasion and may provide valuable insights on the host-parasite interaction.

The last but perhaps most interesting finding is the group protein spots that were matched to the EST of different-stage larvae of *A. cantonensis*, however, they could not be previously identified. For example, spot 1044, was both found up-regulated in FL5 relative to ML5 and in MA relative to ML5. The catalog of those spots reflects differences in protein expression at different developmental stages. Our study is the first indication for their potential roles in the developmental stages.

## Conclusions

In this work, we used DIGE and MALDI MS/MS techniques to characterize the differentially expressed proteins of various stages of *A. cantonensis* worms. Some of the proteins were found to have important functions that probably impact the survival and development of *A. cantonensis*. Several important features of these proteomes were limited due to the scarcity of genomic information. However, this was the case for the dominant proteins found to differ by gender and developmental stage. Two genes corresponding to the protein spots designated 609, 1004 and the internal control 18s were chosen for quantitative real-time PCR (qPCR) analysis to quantify their transcript levels, and the qPCR results were consistent with those of the DIGE studies. These findings will provide a new basis for understanding the developmental mechanism of *A. cantonensis* and the host–parasite relationship. They may also assist searches for candidate proteins suitable for use in diagnostic assays and as drug targets for the control of eosinophilic meningitis induced by *A. cantonensis*. The details of the mechanism underlying their action require further study.

## Supporting Information

File S1 **Includes the following tables: Table S1.** Protein spots of scan profile. **Table S2**. Significant differences of protein spots and their pairwise fold changes. **Table S3**. Significant differences of protein spots and their pairwise fold changes between FA/FL5 and MA/ML5 from L3, respectively. **Table S4**. Peptides Information of unidentified protein spots match to the EST of *A. cantonensis*.
(RAR)
